# CROSS-DISCIPLINE COLLABORATIVE INTERNSHIPS TO REDUCE FEAR OF FALLING CONTRIBUTE TO AGE-FRIENDLY INITIATIVES

**DOI:** 10.1093/geroni/igac059.207

**Published:** 2022-12-20

**Authors:** Andrea June, Allison Seifert

**Affiliations:** Central Connecticut State University, New Britain, Connecticut, United States; Central Connecticut State University, New Britain, Connecticut, United States

## Abstract

The Age Friendly University (AFU) initiative provides a vision to inspire institutions of higher education to respond to the interests and needs of an aging population to promote well-being and quality of life. At Central Connecticut State University (CCSU), we have utilized multiple strategies to engage collaborators within our university and our community to further age-inclusive efforts. The purpose of this presentation is to describe the ongoing collaboration, through the Matter of Balance program, with the Connecticut Healthy Living Collective, an emerging hub of state, regional and local agencies and organizations dedicated to healthy aging in communities throughout our state. The Matter of Balance program is an empirically supported community-based treatment to reduce fear of falling among older adults. We will first discuss the pilot internship program offered within the Department of Psychological Science that trained students as program facilitators which was cut short in Spring 2020 due to COVID 19. We will then describe “Version 2.0” of the collaboration which was offered in the Spring 2022 semester. This time, it was grounded in a gerontology internship course that expanded to include research, faculty, and student collaboration with the Department of Physical Education and Human Performance. In addition to providing these overall descriptions, the presenters will review the strengths and challenges of each approach, share results about the students’ experiences, and highlight the multidimensional value this intentional effort offers.

